# Clinical potential of computed tomography-guided coaxial needle sequence puncture with suture-anchored needle guidance and coupled needle retraction-and-advancement technique biopsy of pulmonary nodules

**DOI:** 10.3389/fonc.2026.1762515

**Published:** 2026-04-01

**Authors:** Shan Song, Chao Zhou, Jie Tan, Wenxia Hu, Yinhu Wang, Lihua Ma, Yun Wang, Si Li, Fei Peng, Wuzhuang Sun

**Affiliations:** 1 Department of Pneumology, The Fourth Hospital of Hebei Medical University, Shijiazhuang, Hebei, China; 2 Department of Anesthesiology, The Fourth Hospital of Hebei Medical University, Shijiazhuang, Hebei, China; 3 Department of Pneumology, Shijiazhuang Traditional Chinese Medicine Hospital, Shijiazhuang, Hebei, China; 4 Department of Pneumology, The First Hospital of Hebei Medical University, Shijiazhuang, Hebei, China

**Keywords:** accurate puncture, biopsy needle, coaxial needle, CT-guided, small pulmonary nodule

## Abstract

**Introduction:**

Computed tomography (CT)-guided percutaneous pulmonary nodule biopsy is a technique for determining the nature of nodules. However, the puncture accuracy and biopsy success rate decline as pulmonary nodule diameter decreases. This study aimed to evaluate the clinical applicability of a CT-guided coaxial needle using suture-anchored needle guidance sequential puncture and coupled needle retraction-and-advancement technique biopsy modes for pulmonary nodules, compared with conventional pulmonary nodule synchronous puncture and coaxial needle insertion biopsy modes. In addition, pulmonary nodule puncture accuracy and biopsy success rates were evaluated.

**Methods:**

A total of 158 patients with solitary pulmonary nodules were randomly assigned to two groups (79 patients per group). Group A underwent a sequential puncture mode of pulmonary nodules and coupled needle retraction-and-advancement technique biopsy, whereas Group B underwent a conventional synchronous puncture mode of pulmonary nodules and coaxial needle insertion biopsy (Group B). The first coaxial needle withdrawal rate, off-target rate, target periphery rate, target central rate, biopsy tissue strip length, first and final biopsy success rates of pulmonary nodules, and procedure-related complications were compared between the groups.

**Results:**

The success rate of first-attempt biopsy in Group A was significantly higher than that in Group B (76/79 [96.2%] vs. 60/79 [75.9%], *P* = 0.002). The relative risk (RR) of first-attempt biopsy success in Group A vs. Group B was 1.27 (95% confidence interval [CI], 1.13–1.43%), and the absolute risk difference was 20.3% (95% CI, 8.7–31.9%). Needle withdrawal occurred in 4 patients (5.1%) in Group A, which was significantly lower than the 31 (39.2%) patients in Group B (*P* < 0.001). The composition ratios of the three spatial positions of coaxial needle tips and pulmonary nodules differed significantly between the two groups (*P* < 0.01). The ratio of sampling strip length to pulmonary nodule diameter × 100% on the first biopsy was significantly higher in Group A than in Group B (100 [50,100] vs. 40 [3,76], *P* < 0.001).

**Discussion:**

CT-guided coaxial needle sequential puncture with suture-anchored needle guidance can enhance pulmonary nodule puncture accuracy, and the coupled needle retraction-and-advancement technique improves biopsy success rate.

**Clinical trial registration:**

http://www.chictr.org.cn, identifier (ChiCTR2400088764).

## 1 Introduction

Computed tomography (CT)-guided percutaneous pulmonary nodule biopsy is a widely used technique for determining the nature of the nodules (1, 2). However, puncture accuracy and biopsy success rate of pulmonary nodules decline as the diameter of pulmonary nodules decreases (3, 4). Various approaches to pulmonary nodule puncture and biopsy have been explored (4–13). Nonetheless, the traditional coaxial needle puncture method has the following shortcomings:1) For pulmonary nodules with a diameter of ≤1 cm or adjacent to the diaphragm and blood vessels, real-time guidance cannot be achieved during lung puncture, and positioning deviation is likely to occur due to respiratory movement; 2) Nodules near the diaphragm are significantly affected by respiratory movement. Even with respiratory control techniques (such as breath-holding puncture), the risk of pneumothorax or bleeding may still increase due to displacement; 3) Repeated adjustments of the angle or depth of the coaxial needle may expand pleural damage (14, 15). The incidence of pneumothorax can reach 15–28% (especially in patients with emphysema) (14, 16, 17); and 4) If blood vessels are not accurately avoided during the operation, the coaxial sheath may puncture small blood vessels, leading to hemoptysis or intrathoracic hemorrhage. Among these challenges, improving the accuracy and success rate of lung nodule biopsy remains an urgent problem to be solved in clinical practice (18).

Previous studies have shown that the suture-anchored needle guidance method can more accurately control the puncture angle and depth, effectively improve puncture precision, and significantly reduce the risk of needle tract bleeding (19). In this study, a sequential pulmonary nodule puncture mode and a coupled needle (coaxial and biopsy needles) retraction-and-advancement biopsy method were employed and compared with the conventional synchronous pulmonary nodule puncture mode and coaxial needle insertion biopsy method to evaluate their clinical applicability. This study aimed to determine whether these combined methods improve the puncture accuracy and biopsy success rate of pulmonary nodules.

## 2 Materials and methods

### 2.1 Study patients and data collection

**Figure 1:**
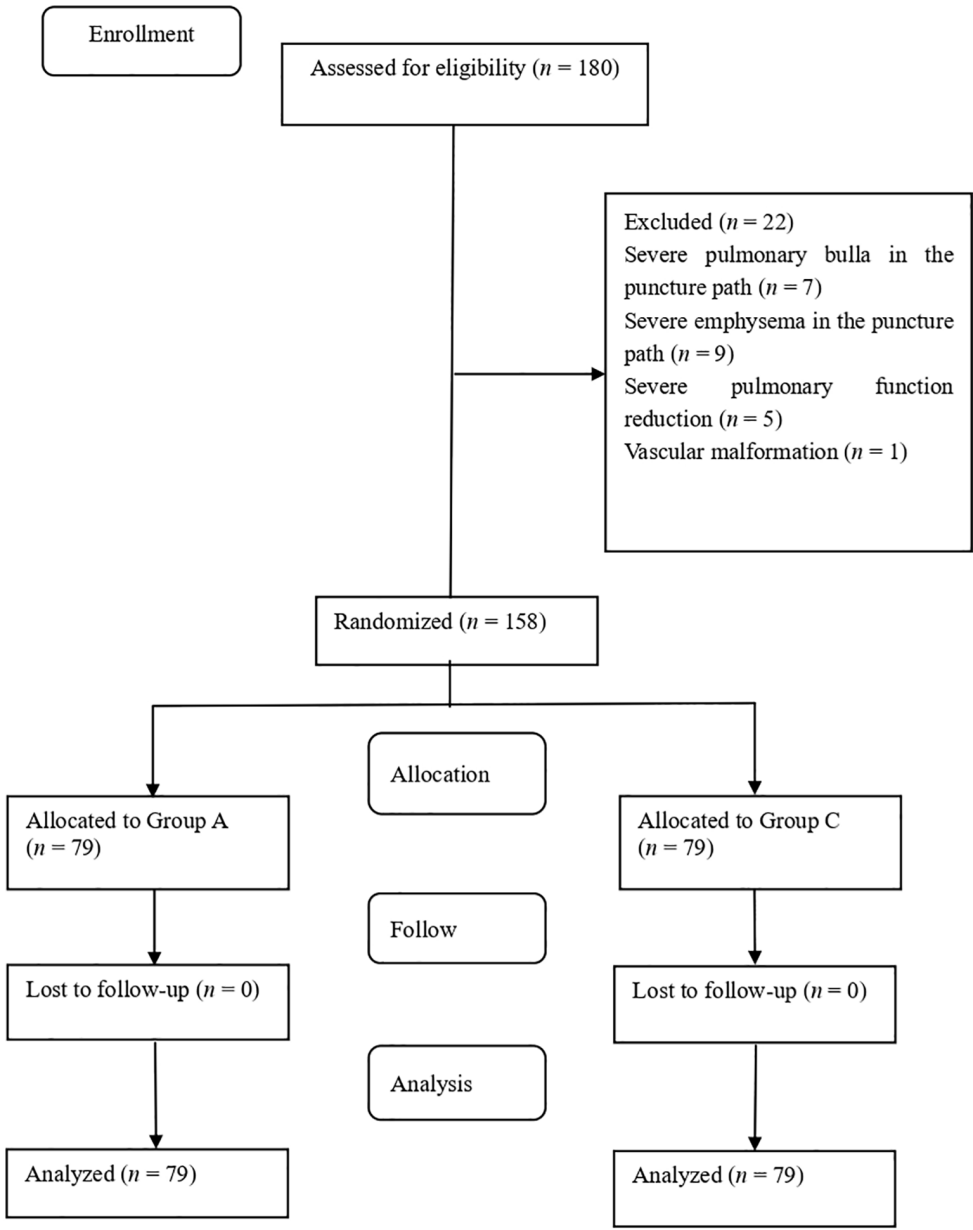
Flow diagram of the study.

This was a randomized controlled single-center clinical trial. This study was approved by the Ethics Committee of Shijiazhuang Traditional Chinese Medicine (TCM) Hospital on September 12, 2023 (ID: 20230912001), and registered with the China Clinical Trial Registration Center on August 31, 2024 (http://www.chictr.org.cn; registration number: ChiCTR2400088764). This trial began on September 2, 2024, and ended on April 30, 2025. All patients signed an informed consent form before the trial. The Consolidated Standards of Reporting Trials (CONSORT) checklist was used for patient enrolment and allocation (**Figure 1**). Patients with solitary pulmonary nodules admitted to Shijiazhuang TCM Hospital between September 2, 2024, and April 30, 2025, were selected. This manuscript is prepared in accordance with the CONSORT reporting checklist.

The authors are accountable for all aspects of the work in ensuring that questions related to the accuracy or integrity of any part of the work are appropriately investigated and resolved. This study was conducted in accordance with the principles of the Declaration of Helsinki and its subsequent amendments. All participants were informed of the procedural risks and provided a written informed consent form.

The inclusion criteria were as follows: 1) Lung nodule diameter ranging from 5 to 15 mm; 2) CT of the nodule density showing solid pulmonary nodules, mixed ground-glass nodules (mGGN), and pure ground-glass nodules (pGGN); 3) prothrombin time-to-international normalized ratio< 1.5 and platelet count > 50×10^9^/L; 4) patients agreeing to participate in the study and signing the informed consent form; and 5) anticoagulant discontinuation at least 7 days before procedure.

The exclusion criteria were as follows: 1) severe cardiopulmonary insufficiency (such as severe pulmonary hypertension), 2) uncorrectable coagulation dysfunction, 3) anatomically or functionally isolated lung, 4) obvious infectious lesions on the puncture path, 5) bullae, vascular malformation, aneurysm, or pulmonary hydatid disease, 6) mechanically ventilated patients, and 7) disturbance of consciousness, dysphoria, or severe cough.

Upon enrollment, all patients were randomly assigned to two groups with 79 patients in each group, using a 1:1 allocation ratio. A statistician who was not involved in patient enrollment and data collection generated the randomization sequence using IBM SPSS 21.0 software. Allocation concealment was implemented using sequentially numbered, opaque, and sealed envelopes. Envelopes were made of light-impermeable kraft paper, sealed with glue, stamped with the study-specific seal, and only labeled with a consecutive number (1–158) without group information. An independent research assistant opened the envelopes sequentially after enrollment to determine the group assignment. Each envelope contained a card indicating the group assignment (Group A or Group B) and was opened only after the patient had been enrolled and baseline data collected by an independent research assistant not involved in the randomization process. Outcome assessors (pathologists) were blinded to group allocation. All biopsy specimens were labeled with unique identification numbers (assigned by an independent research assistant) without any group-related information. Pathologists only received basic clinical data (age, sex, nodule size, and specimens with unique numbers) and judged the biopsy success based on a unified pathological standard. Communication between pathologists and researchers was limited to specimen numbers to maintain blinding until data locking. Group A involved the sequential puncture mode and coupled needle retraction-and-advancement technique biopsy mode for pulmonary nodules, and Group B included the synchronous puncture mode and coaxial needle insertion biopsy method for pulmonary nodules.

### 2.2 Instruments and methods

A 64-slice spiral CT machine (General Electric, Boston, Massachusetts, USA) was used in this study. TSK Co. Ltd. (Kyoto, Japan) provided the disposable coaxial puncture positioning needle (coaxial needle; 17G×6 cm and 17G×12 cm) and a semi-automatic tissue cutting biopsy needle (biopsy needle; 18G×8.5 cm and 18G×14.5 cm). A biopsy kit, surgical hole towel, 100-cm white silk thread, several surgical forceps, 2% lidocaine, a monitor, and oxygen inhalation equipment were also provided (TSK Co. Ltd.). Preoperative routine blood tests, coagulation tests, cardiopulmonary function tests, and enhanced lung CT scans were performed to exclude contraindications for lung puncture.

The appropriate body position was selected based on the location of the pulmonary nodule, aiming for participant comfort and ease of operation. CT scanning parameters included a tube voltage of 120 kV, tube current of 200–300 mA, slice thickness of 2.5 mm, and reconstructed slice thickness of 1.25 mm. The participants were instructed to breathe calmly. The operator referred to the enhanced lung CT scan, selected the optimal puncture point, placed the positioning grid on the chest surface, and determined the puncture point, direction, and depth from the skin to the nodule under CT guidance.

**Figure 2:**
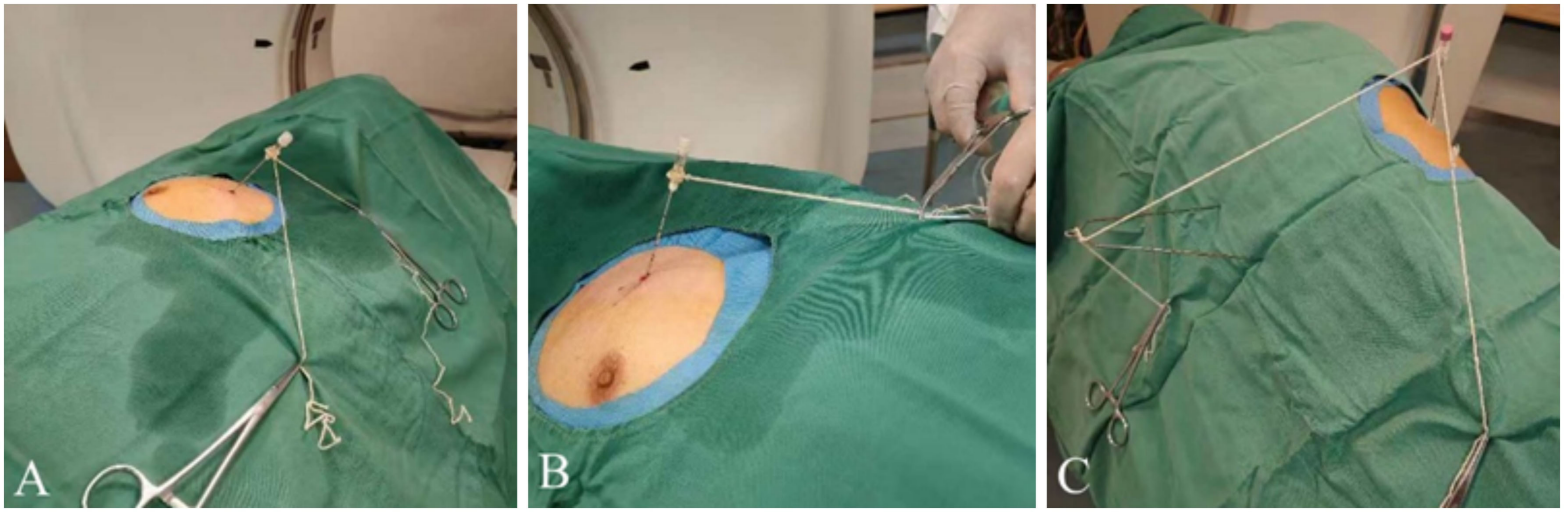
Schematic of the coaxial suture-anchored needle guidance method. **(A)** Double-threaded suture-anchored needle guidance; **(B)** Single-threaded suture-anchored needle guidance; **(C)** single-support-assisted suture-anchored needle guidance. **(A, B)** are mostly used for coaxial needle vertical CT scanning beds to puncture pulmonary nodules, while **(C)** is suitable for coaxial needle parallel CT scanning beds or parallel to the tangent line of the pleura. CT, computed tomography.

**Figure 3:**
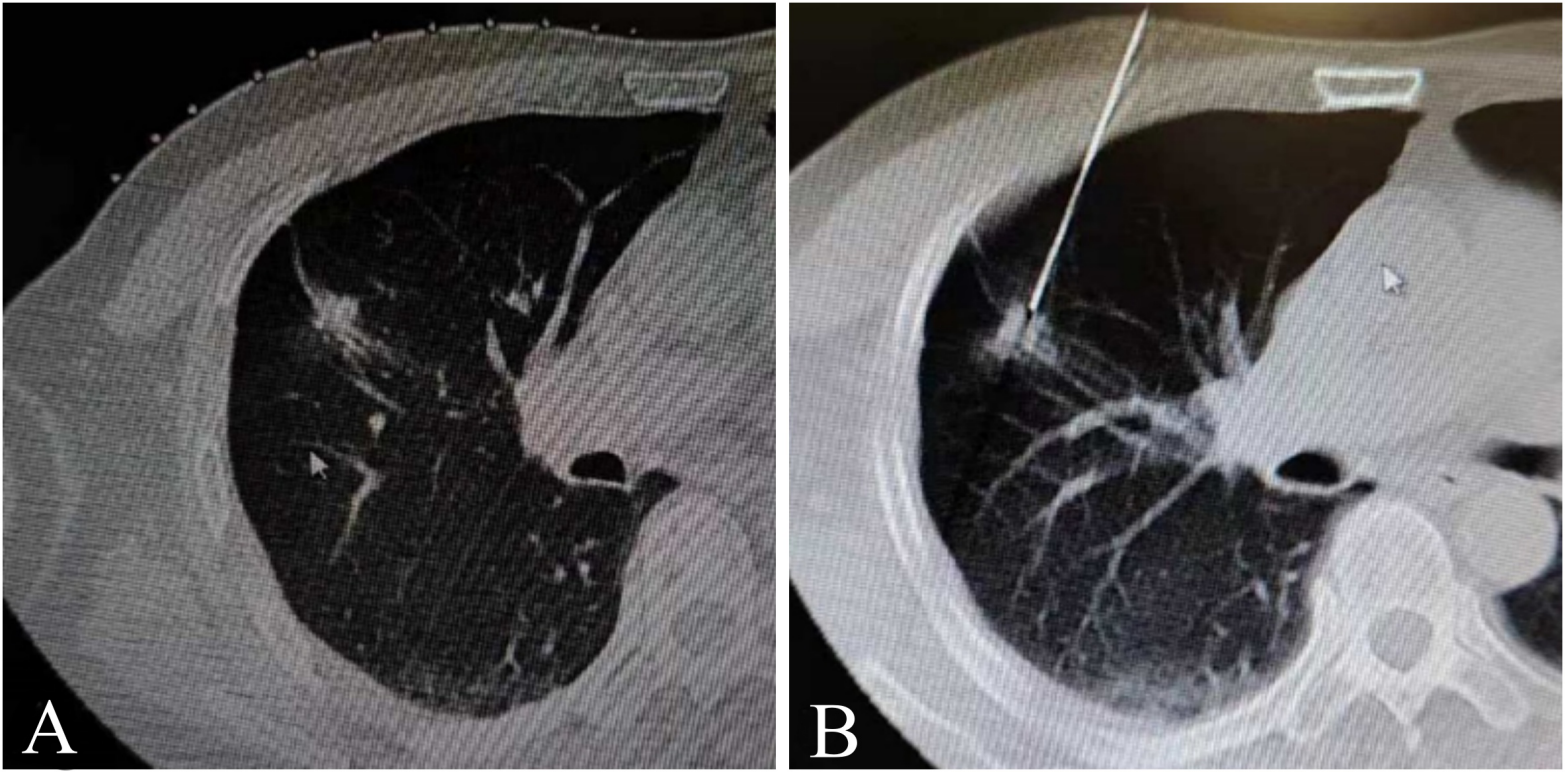
A 63-year-old male with a standard lung window revealed a small solid nodule under the subpleural region of the right middle lobe. Pathological results showed lung adenocarcinoma. **(A)** Plain chest computed tomography (CT) scan showing a 13-mm solid nodule under the subpleural area of the right middle lobe; **(B)** Puncture of pulmonary nodule with a coaxial needle assisted by suture-anchored guidance, and the needle tip inserted into the center of the nodule.

**Figure 4:**
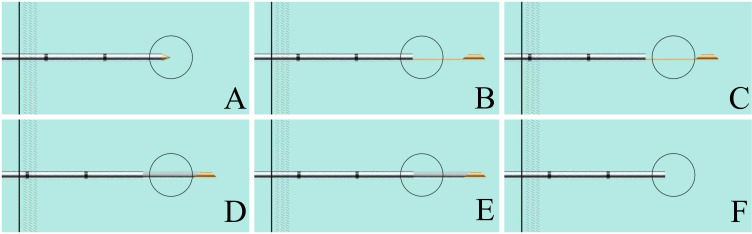
Schematic of coupled needle (coaxial and biopsy needle) retraction-and-advancement technique for biopsy of pulmonary nodules. **(A)** Coaxial needle inserted into the pulmonary nodule; **(B)** Cutting groove penetrating pulmonary nodules; **(C)** Coupled needle fixed and lifted together, exposing the whole nodule diameter to the cutting groove; **(D)** Firing biopsy needle; **(E)** Coupled needle fixed and inserted downward reset; **(F)** Withdrawing the biopsy needle and removing the nodule tissue.

Regarding Group A, the suture-anchored method of the coaxial needle tail was adopted to assist in coaxial needle pulmonary nodule puncture; the middle of the white thread was fixed to the coaxial needle tail, and both ends of the thread were secured with vascular clamps on the surface of the hole towel (**Figure 2**). First, the direction of the needle tip was changed and pointed to the lung nodules by adjusting the pulling direction and tension of both ends of the thread, according to the spatial position of the needle tip and pulmonary nodules shown on the CT scan. Subsequently, the needle body was pushed forward by first adjusting the direction of the needle and then advancing it in the forward-sequential puncture mode (**Figure 3**). After inserting the coaxial needle into the center of the pulmonary nodule, the needle core was withdrawn, and a biopsy needle was inserted along the outer sheath of the coaxial needle to make the cutting groove exceed the front end of the outer sheath by 0.5–1.5 cm. After fixing both needles together, they were lifted by 0.5–1.5 cm simultaneously, exposing the diameter of the pulmonary nodule completely in the biopsy slot. The biopsy needle was then fired, and both needles were fixed and inserted downward synchronously, keeping the outer sheath in the center of the pulmonary nodule. The biopsy needle was withdrawn, and the biopsy tissue was collected. In this manner, the circular process of fixing both needles, simultaneous lifting and cutting, concurrent insertion down reset, and sampling was repeated (**Figure 4**).

Regarding Group B, using the current routine manual puncture mode and biopsy method for pulmonary nodules, a coaxial needle was inserted through the puncture point and along the direction of the puncture. Based on the CT scan showing the spatial location of the needle tip and the pulmonary nodule, the direction of the needle tip was adjusted while advancing the needle forward. A coaxial needle tip was inserted into the pulmonary nodule to ensure that the front end of the sheath remained approximately 2–3 mm in the superficial layer of the nodule. The needle core was subsequently withdrawn, and a biopsy needle was inserted along the coaxial needle sheath for pulmonary nodule biopsy. Three pulmonologists with over 11 years of experience in lung biopsies performed all procedures.

### 2.3 Outcomes

The primary outcome was the success rate of biopsy on the first attempt. A successful biopsy of the pulmonary nodule was defined as the presence of abnormal lung tissue on histopathological examination; otherwise, the biopsy failed. The secondary outcomes were the number of needle withdrawals, the spatial position of the needle tip and pulmonary nodule, sampling strip length divided by pulmonary nodule diameter × 100% on the first biopsy, the success rate of biopsy on the entire attempt, and the number of pneumothorax and hemorrhages.

During the puncture procedure, any instance of coaxial needle withdrawal ≥1 was considered a needle withdrawal event. The spatial relationship between the needle tip and the pulmonary nodule was recorded upon initial insertion, which was defined as the needle tip deviating from the nodule; the target peripheral position indicated that the needle tip was located in the outer third of the three-ring target area of the nodule (outer ring), and the target central position denoted that the needle tip was located in the middle and inner third of the three-ring target area of the nodule (central ring).

### 2.4 Statistical analysis

Based on our clinical experience, the success rate of biopsy on the first attempt in conventional pulmonary nodule synchronous puncture and coaxial needle insertion biopsy modes was 60%. In our pilot study, the success rate of biopsy on the first attempt using a CT-guided coaxial needle with suture-anchored needle guidance and sequential puncture and coupled needle retraction-and-advancement technique biopsy modes for pulmonary nodules was 81%. According to the following comparison formula of the two independent sample rates:


$$n=0.5\times {\left[\frac{\left(u_{\alpha }+u_{\beta }\right)}{\sin^{-1}\sqrt{p_{1}}-\sin^{-1}\sqrt{p_{2}}}\right]}^{2}$$

Each group requires 72 participants (power: 0.8; type I error: 0.05; $u_{\alpha }$ = 1.96, $u_{\beta }$ = 0.842). To prevent unexpected situations, such as exclusion and loss, we increased the number of patients in each group by 10%, resulting in 79 patients in each group.

Statistical analyses were performed using the IBM SPSS Statistics for Windows, version 21.0 (IBM Corp., Armonk, NY, USA). The primary outcome was analyzed using the chi-square (χ²) test and expressed as percentages. For the secondary outcomes, categorical variables were analyzed using the χ² test and expressed as percentages; the spatial position of the needle tip and pulmonary nodule was analyzed using the Cochran–Armitage trend test and expressed as percentages. The sampling strip length divided by pulmonary nodule diameter × 100% on the first biopsy was analyzed using a non-parametric test, expressed as median (range).

## 3 Results

### 3.1 Patients and nodule characteristics

Overall, 180 patients were screened for this study, 22 of whom were excluded because they met the exclusion criteria. Consequently, 158 patients with solitary pulmonary nodules were included and categorized into two groups, groups A and B, with 79 patients in each group. The baseline characteristics of the patients and nodules in both groups are presented in **Table 1** and were comparable between the two groups.

**Table 1:** Baseline characteristics of patients and pulmonary nodules for both groups.

Characteristic	Group A (*n* = 79)	Group B (*n* = 79)	*P* value
Sex (*n* [%])			0.873
Male	44 (55.7)	42 (53.1)	
Female	35 (44.3)	37 (46.9)	
Age (years) (mean ± SD [range])	62.0 ± 9.6 (29–85)	62.8 ± 9.5 (30–87)	0.577
Age (*n* [%])			1.000
<65	47 (59.5)	48 (60.8)	
≥65	32 (40.5)	31 (39.2)	
Nodule size (mm) (mean ± SD)	11.2 ± 2.9	10.8 ± 2.9	0.414
Nodule size range (*n* [%])			0.496
5–9 (mm)	23 (29.1)	28 (35.4)	
10–15 (mm)	56 (70.9)	51 (64.6)	
Nodule density (*n* [%])			0.809
sPN	39 (49.4)	35 (44.3)	
mGGN	24 (30.4)	27 (34.2)	
pGGN	16 (20.2)	17 (21.5)	
Distance from pleura to nodule (cm) (*n* [%])			0.758
≤1	15 (19.0)	16 (20.3)	
>1–5	43 (54.4)	46 (58.2)	
>5	21 (26.6)	17 (21.5)	
Basic pulmonary diseases (*n* [%])			0.856
COPD	10 (12.7)	6 (7.6)	
Old tuberculosis	5 (6.3)	7 (8.9)	
Asthma	6 (7.6)	7 (8.9)	
Pulmonary fibrosis	5 (6.3)	4 (5.0)	
Silicosis	1 (1.3)	1 (1.3)	
Asbestosis	1 (1.3)	0	

sPN, solid pulmonary nodules; mGGN, mixed ground-glass nodules; pGGN, pure ground-glass nodules; COPD, chronic obstructive pulmonary disease.

a Two independent sample *t*-tests.

### 3.2 Primary outcome

The success rate of biopsy on the first attempt in Group A was significantly higher than that in Group B ([76, 96.2%] vs. [60,75.9%], *P* = 0.002; **Table 2**). The relative risk (RR) of first-attempt biopsy success in Group A compared with Group B was 1.27 (95% confidence interval [CI], 1.13–1.43%), and the absolute risk difference was 20.3% (95% CI, 8.7–31.9%) (**Table 2**).

**Table 2:** The success rate of biopsy on the first attempt.

*Primary Outcome*	Group A(n=79)	Group B(n=79)	Difference (95% CI)	Relative Risk (95% CI)	*P* value
The success rate of biopsy on the first attempt	76 (96.2%)	60 (75.9%)	20.3% (8.7%–31.9%)	1.27 (1.13–1.43)	<0.001

### 3.3 Secondary outcomes

Four (5.1%) patients with needle withdrawal in Group A were observed, which is significantly lower than that in Group B with 31 (39.2%) patients (*P* < 0.001, **Table 3**). Statistical analysis of the spatial positions (off-target, target peripheral, and target central) revealed that Group A exhibited a predominant trend of a high proportion in the target central position, whereas Group B showed a higher proportion of off-target or target peripheral positions (*P* < 0.01, **Table 3**). The sampling strip length divided by pulmonary nodule diameter × 100% on the first biopsy was significantly higher in Group A than in Group B (100 [50,100] vs. 40 [3,76], *P* < 0.001). All 79 (100%) patients in Group A underwent successful biopsy during the whole attempt, compared with 74 (93.7%) in Group B (*P* = 0.059, **Table 3**). The number of participants with pneumothorax was 16 (20.3%) and 19 (24.0%) in Groups A and B, respectively, and no significant differences were observed (*P* = 0.702, **Table 3**). No significant difference was noted in the bleeding composition ratio between Groups A and B (*P* = 0.576, **Table 3**). In this study, the incidences of pneumothorax and pulmonary hemorrhage in the two groups were low.

**Table 3:** The secondary outcomes in the two groups.

Secondary Outcome	Group A (*n* = 79)	Group B (*n* = 79)	*P* value
Withdrawal (*n* [%])	4 (5.1)	31 (39.2)	<0.001
The spatial position of the needle tip and pulmonary nodule (*n* [%])			
Off-target	2 (2.5)	18 (22.8)	<0.001
Target peripheral	11 (13.9)	23 (29.1)	
Target central	66 (83.5)	38 (48.1)	
The sampling strip length/pulmonary nodule’s diameter *100% on first biopsy (median [range])	100 (50,100)	40 (3,76)	<0.001
The success rate of biopsy on the whole attempt (*n* [%])	79 (100)	74 (93.7)	0.059
Pneumothorax (*n* [%])	16 (20.3)	19 (24.0)	0.702
Hemorrhage (*n* [%])			0.576
Needle path	6 (7.6)	11 (13.9)	
Biopsy area	13(16.5)	10 (12.6)	
Both have	5 (6.3)	4 (5.1)	
None	55 (69.6%)	54 (68.4%)	

Collectively, these results demonstrate that compared with the conventional synchronous puncture and coaxial needle insertion biopsy (Group B), the CT-guided coaxial needle sequence puncture with suture-anchored needle guidance combined with coupled needle retraction-and-advancement technique (Group A) achieves superior performance in improving first biopsy success rate, reducing needle withdrawal rate, enhancing puncture targeting accuracy, and increasing relative sampling length, while maintaining comparable safety profiles regarding pneumothorax and hemorrhage incidence.

## 4 Discussion

With the popularization of early lung cancer screening, the detection of lung nodules, some of which are early lung cancer and precancerous lesions, has increased significantly. CT-guided percutaneous biopsy is the primary method used to determine the nature of the nodules. However, with a gradual reduction in the size of pulmonary nodules, the puncture accuracy and biopsy success rate decrease. The *Chinese Expert Consensus on Early Lung Cancer Diagnosis* (20) states that percutaneous lung biopsy is not recommended for pulmonary nodules with a diameter of<10 mm. When the lesion was<10 mm, the false-negative rate of puncture increased significantly. One of the reasons for this dilemma is that the current routine puncture mode and biopsy method for pulmonary nodules limit the improvement of puncture accuracy and biopsy success rate of lung nodules. Exploring new puncture modes and biopsy methods for pulmonary nodules may improve the puncture accuracy and biopsy success rate of pulmonary nodules and resolve this dilemma.

Our study demonstrated that Group A (sequential puncture with suture-anchored guidance and coupled needle retraction-and-advancement biopsy) had a significantly lower needle withdrawal rate (5.1% vs. 39.2%, *P* < 0.001) and a higher proportion of target-central punctures (83.5% vs. 48.1%, *P<* 0.01) than Group B (conventional synchronous puncture and coaxial needle insertion biopsy). These results indicate that sequential puncture achieves higher puncture accuracy than synchronous puncture.

The core mechanism underlying this advantage lies in mitigating the impact of internal stress. CT-guided coaxial needle puncture is inherently a semi-blind procedure: adjusting the needle angle while advancing (synchronous puncture) generates dynamic and static internal stress in surrounding tissues, which can displace the needle tip when released. Respiratory motion exacerbates this issue—lung nodules move an average of 1.76 cm during calm breathing (21), and diaphragm movement (2–4 cm) induces longitudinal stress that causes the needle to swing. Additionally, the heterogeneous elasticity of the chest wall and lung tissue amplifies needle deviation via lever effects, particularly for deeper or obliquely placed needles. In Group A, fixing the coaxial needle tail to the surgical drape with silk threads applies a constant external force, stabilizing the needle and immobilizing adjacent lung tissue. This substantially reduces needle swing caused by internal stress, ensuring consistent spatial alignment between the needle tip and nodule during CT guidance and puncture. By separating needle adjustment (via thread tension/direction) from advancement (sequential puncture), operators gain objective reference points, reducing reliance on experience and improving targeting accuracy.

Furthermore, Group A achieved a significantly higher first-pass biopsy success rate (96.2% vs. 75.9%, *P* = 0.002) and a larger sampling ratio (100 [50,100]% vs. 40 [3,76]%, *P* < 0.001) than Group B. The coupled needle retraction-and-advancement technique addresses limitations of conventional biopsy methods: inserting the coaxial needle into the nodule center locks the target, preventing internal stress-induced displacement. Lifting both needles to expose the full nodule diameter in the biopsy slot ensures adequate tissue sampling—critical for small or ground-glass nodules (mGGN/pGGN), where partial sampling frequently leads to diagnostic failure. Notably, Group B’s overall biopsy success rate (93.7%) approached that of Group A (100%) with multiple attempts (*P* = 0.059), a finding of borderline statistical significance. This suggests that repeated passes can compensate for initial low success rates in conventional techniques; however, our sequential puncture and coupled biopsy method reduces the number of necessary passes. Clinically, this minimizes pleural damage, shortens procedure time, and lowers the risk of cumulative complications—key advantages for patients, particularly those with fragile lung function or coagulation disorders.

The primary reason why multiple attempts in Group B partially compensated for the initial low success rate is closely related to the technical characteristics of conventional synchronous puncture and biopsy. First, for Group B, repeated needle adjustments and reinsertions during multiple passes enabled operators to continuously correct the puncture direction based on real-time CT feedback, offsetting the positioning deviation caused by internal stress to a certain extent. Second, multiple biopsies increased the probability of sampling effective tissue—particularly for larger nodules (10–15 mm), where even partial sampling in the peripheral area could provide abnormal tissue sufficient for pathological diagnosis. However, this “compensation effect” has clear limitations: repeated punctures increase the risk of pleural damage and bleeding (though not statistically significant in this study), prolong the operation time, and augment the patient’s physical and psychological burden. In contrast, Group A achieved a 96.2% success rate with a single complete attempt, relying on the high positioning accuracy of suture-anchored sequential puncture and the sufficient tissue sampling of the coupled needle retraction-and-advancement technique. This highlights the core clinical value of our technique: while ensuring final diagnostic success, it significantly reduces the number of puncture passes, shortens operation time, and minimizes iatrogenic damage—particularly important for fragile patients (including the elderly or those with underlying lung diseases) or small nodules (5–9 mm) where repeated punctures carry higher risks.

Our first-pass success rate of 96.2% for nodules ≤15 mm (including 29.1% of nodules 5–9 mm) is superior to results reported in recent studies of small nodule biopsy techniques. Jeon et al. (11) used laser guidance for CT-guided biopsy and achieved a first-pass success rate of 78.3% for small nodules, with technical complexity and equipment costs limiting widespread adoption. Zhang et al. (23, 24) reported a 89.2% first-pass accuracy using three-dimensional (3D)-printed templates; however, template fabrication requires 3D modeling and printing facilities, extends preparation time (2–3 days), and increases costs (≈$500–$800 per template). In contrast, our suture-anchored guidance uses standard clinical supplies (silk thread and vascular clamps) with no additional equipment costs. The procedure adds<5 min to the total operation time, compared to 15–20 min for laser calibration or 3D template positioning. Tsai et al. (22) described a sterile drape-supported sequential puncture method with a first-pass success rate of 82.1%; conversely, this technique lacked stable thread fixation, leading to inconsistent needle control. Our dual-thread and stent-assisted modifications address this shortcoming, improving targeting precision without compromising accessibility.

A key strength of our technique is its real-world applicability, particularly in community hospitals and resource-limited settings. In contrast to laser guidance or 3D printing—which require specialized equipment, trained technicians, and significant capital investment—our method leverages readily available supplies and can be mastered by pulmonologists with standard CT-guided biopsy experience (≥5 years). This scalability is critical considering the global disparity in access to advanced diagnostic tools: in low- and middle-income countries, >70% of hospitals lack 3D printing capabilities or laser guidance systems (25). For small nodules adjacent to the diaphragm or blood vessels—where respiratory motion and anatomical constraints increase puncture difficulty—our technique’s stress-mitigating design outperforms conventional methods without the need for expensive navigation technology. Additionally, the reduced number of needle passes lowers the risk of pneumothorax and hemorrhage in high-risk patients (e.g., those with chronic obstructive pulmonary disease or pulmonary fibrosis), expanding the scope of eligible candidates for biopsy.

Pneumothorax and pulmonary hemorrhage are common complications of percutaneous lung puncture and biopsy (22). No significant difference was observed in the incidence of complications between Groups A and B. The CT-guided coaxial needle with suture-anchored needle guidance sequential puncture and coupled needle retraction-and-advancement technique biopsy modes for pulmonary nodules did not increase the risk of pneumothorax or pulmonary hemorrhage.

This study had some limitations. First, the small sample size (158 patients) and single-center design may limit the generalizability of our results; multi-center trials with larger cohorts are needed to validate these findings. Second, the coupled needle retraction-and-advancement technique causes slight local lung tissue trauma and requires moderate procedural proficiency, which may affect learning curves for novice operators. Third, the absence of long-term follow-up or surgical resection as a gold standard prevents calculation of diagnostic sensitivity, specificity, and negative predictive value. Our definition of “successful biopsy” (obtaining abnormal tissue) does not account for false negatives—a critical gap, as small nodules (especially pGGNs) may have heterogeneous pathology, and inadequate sampling could miss malignant foci. Future studies should include 12–24 months of clinical and imaging follow-up, or correlate biopsy results with surgical pathology, to determine the true diagnostic accuracy of the technique. Finally, we did not assess cost-effectiveness quantitatively; future research should compare procedure costs, complication management expenses, and resource utilization between our technique and advanced modalities.

In conclusion, conventional synchronous puncture and coaxial needle insertion are limited by internal stress-induced needle deviation, making them suboptimal for small pulmonary nodules. Our suture-anchored sequential puncture mitigates internal stress to improve puncture accuracy, whereas the coupled needle retraction-and-advancement technique enhances biopsy yield. Although multiple attempts can compensate for initial failures in conventional techniques, our approach reduces the number of necessary passes, minimizing patient risk and procedural burden. Addressing the limitation of long-term diagnostic validation is critical to establishing the technique’s role in early lung cancer diagnosis.

## Data Availability

The original contributions presented in the study are included in the article/supplementary material. Further inquiries can be directed to the corresponding author.

## References

[1] Yoon SH Lee SM Park CH Lee JH Kim H Chae KJ . Clinical practice guideline for percutaneous transthoracic needle biopsy of pulmonary lesions: A consensus statement and recommendations of the Korean Society of Thoracic Radiology. Korean J Radiol. (2021) 22:263–80. doi: 10.3348/kjr.2020.0137. PMID: 33236542 PMC7817630

[2] Macmahon H Naidich DP Goo JM Lee KS Leung ANC Mayo JR . Guidelines for management of incidental pulmonary nodules detected on CT images: From the Fleischner Society 2017. Radiology. (2017) 284:228–43. doi: 10.1148/radiol.2017161659. PMID: 28240562

[3] Li Y Yang CF Peng J Li B Zhang C Yu JH . Small (≤ 20 mm) ground-glass opacity pulmonary lesions: Which factors influence the diagnostic accuracy of CT-guided percutaneous core needle biopsy? BMC Pulm Med. (2022) 22:265. doi: 10.1186/s12890-022-02058-z. PMID: 35799223 PMC9264544

[4] Hwang EJ Kim H Park CM . Cone beam computed tomography virtual navigation-guided transthoracic biopsy of small (≤ 1 cm) pulmonary nodules: Impact of nodule visibility during real-time fluoroscopy. Br J Radiol. (2018) 91:20170805. doi: 10.1259/bjr.20170805. PMID: 29595322 PMC6221790

[5] Rickets W Lau KKK Pollit V Mealing S Leonard C Mallender P . Exploratory cost-effectiveness model of electromagnetic navigation bronchoscopy (ENB) compared with CT-guided biopsy (TTNA) for diagnosis of Malignant indeterminate peripheral pulmonary nodules. BMJ Open Respir Res. (2020) 7:e000595. doi: 10.1136/bmjresp-2020-000595. PMID: 32796019 PMC7430329

[6] Wu Q Cao B Zheng Y Liang B Liu M Wang L . Feasibility and safety of fine positioning needle-mediated breathing control in CT-guided percutaneous puncture of small lung/liver nodules adjacent to diaphragm. Sci Rep. (2021) 11:3411. doi: 10.1038/s41598-021-83036-z. PMID: 33564042 PMC7873283

[7] Fu R Zhang C Zhang T Chu XP Tang WF Yang XN . A three-dimensional printing navigational template combined with mixed reality technique for localizing pulmonary nodules. Interact Cardiovasc Thorac Surg. (2021) 32:552–9. doi: 10.1093/icvts/ivaa300. PMID: 33751118 PMC8923295

[8] Xu Y Fu J Cao W Zhu L Jin Y Yin Q . Efficacy and safety of new disposable percutaneous positioning device invented to facilitate the precision of percutaneous core needle lung biopsy: A prospective, open and randomized controlled study. J Thorac Dis. (2021) 3:4965–76. doi: 10.21037/jtd-20-3282. PMID: 34527335 PMC8411179

[9] Fontana F Piacentino F Ierardi AM Carrafiello G Coppola A Muollo A . Comparison between CBCT and fusion PET/CT-CBCT guidance for lung biopsies. Cardiovasc Intervent Radiol. (2021) 44:73–9. doi: 10.1007/s00270-020-02613-3. PMID: 32895781

[10] Ji Z Wang G Chen B Zhang Y Zhang L Gao F . Clinical application of planar puncture template-assisted computed tomography-guided percutaneous biopsy for small pulmonary nodules. J Cancer Res Ther. (2018) 14:1632–7. doi: 10.4103/jcrt.JCRT_1017_17. PMID: 30589051

[11] Jeon MC Kim JO Jung SS Park HS Lee JE Moon JY . CT-guided percutaneous transthoracic needle biopsy using the additional laser guidance system by a pulmonologist with 2 years of experience in ct-guided percutaneous transthoracic needle biopsy. Tuberc Respir Dis (Seoul). (2018) 81:330–8. doi: 10.4046/trd.2017.0123. PMID: 29926547 PMC6148095

[12] Tsai SCS Wu TC Lai YL Lin FCF . Preoperative computed tomography-guided pulmonary nodule localization augmented by laser angle guide assembly. J Thorac Dis. (2019) 11:4682–92. doi: 10.21037/jtd.2019.10.60. PMID: 31903257 PMC6940224

[13] Christou AS Amalou A Lee HW Rivera J Li R Kassin MT . Image-guided robotics for standardized and automated biopsy and ablation. Semin Intervent Radiol. (2021) 38:565–75. doi: 10.1055/s-0041-1739164. PMID: 34853503 PMC8612841

[14] Yildirim E Kirbas I Harman A Ozyer U Tore HG Aytekin C . CT-guided cutting needle lung biopsy using modified coaxial technique: factors effecting risk of complications. Eur J Radiol. (2008) 70:57–60. doi: 10.1016/j.ejrad.2008.01.006. PMID: 18294798

[15] Zhao Y Bao D Wu W Tang W Xing G Zhao X . Development and validation of a prediction model of pneumothorax after CT-guided coaxial core needle lung biopsy. Quant Imag Med Surg. (2022) 12:5404–19. doi: 10.21037/qims-22-176. PMID: 36465829 PMC9703113

[16] Saji H Nakamura H Tsuchida T Tsuboi M Kawate N Konaka C . The incidence and the risk of pneumothorax and chest tube placement after percutaneous CT-guided lung biopsy: the angle of the needle trajectory is a novel predictor. Chest. (2002) 121:1521–6. doi: 10.1378/chest.121.5.1521. PMID: 12006438

[17] Huo YR Chan MV Habib AR Lui I Ridley L . Pneumothorax rates in CT-guided lung biopsies: a comprehensive systematic review and meta-analysis of risk factors. Brit J Radiol. (2020) 93:20190866. doi: 10.1259/bjr.20190866. PMID: 31860329 PMC7362905

[18] Guo Z Shi H Li W Lin D Wang C Liu C . Chinese multidisciplinary expert consensus: Guidelines on percutaneous transthoracic needle biopsy. Thorac Cancer. (2018) 9:1530–43. doi: 10.1111/1759-7714.12849. PMID: 30221455 PMC6209790

[19] Tokue H Tsushima Y Ishizaka H Nakazawa A . Use of a novel coaxial guide needle-wire (GNW) combination system for computed tomography guided radiofrequency tumor ablation. World J Surg Oncol. (2011) 9:127. doi: 10.1186/1477-7819-9-127. PMID: 21995771 PMC3204259

[20] Chinese Thoracic Society . Chinese expert consensus on diagnosis of early lung cancer (2023 edition). Zhonghua Jie He Hu Xi Za Zhi. (2023) 46:1–18. doi: 10.1097/cm9.0000000000003055. PMID: 36617923

[21] Chen A Pastis N Furukawa B Silvestri GA . The effect of respiratory motion on pulmonary nodule location during electromagnetic navigation bronchoscopy. Chest. (2015) 147:1275–81. doi: 10.1378/chest.14-1425. PMID: 25357229

[22] Tsai IC Tsai WL Chen MC Chang GC Tzeng WS Chan SW . CT-guided core biopsy of lung lesions: A primer. AJR Am J Roentgenol. (2009) 193:1228–35. doi: 10.2214/AJR.08.2113. PMID: 19843735

[23] Zhang L Li M Li Z Kedeer X Wang L Fan Z . Three-dimensional printing of navigational template in localization of pulmonary nodule: A pilot study. J Thorac Cardiovasc Surg. (2017) 154:2113–2119.e7. doi: 10.1016/j.jtcvs.2017.08.065. PMID: 29017792

[24] Zhang L Wang L Kadeer X Zeyao L Sun X Sun W . Accuracy of a 3-dimensionally printed navigational template for localizing small pulmonary nodules: A noninferiority randomized clinical trial. JAMA Surg. (2019) 154:295–303. doi: 10.1001/jamasurg.2018.4872. PMID: 30586136 PMC6484816

[25] Hu H Li C Lv T Li H Hu Y Shen Q . Contrast-enhanced computed tomography prior to percutaneous transthoracic needle biopsy reduces the incidence of hemorrhage. Ann Transl Med. (2021) 9:288. doi: 10.21037/atm-20-4384. PMID: 33708915 PMC7944326

